# Study on the influence of anti stripping agent on the rheological properties of asphalt at high and low temperatures

**DOI:** 10.1371/journal.pone.0339344

**Published:** 2026-01-30

**Authors:** Qiliang Liu, Fei Zhou, Jun’an Lei, Fujing Zhao

**Affiliations:** 1 School of Civil Engineering, Science and Technology College of Hubei University of Arts and Science, Xiangyang, China; 2 School of Civil Engineering and Architecture, HuBei University of Arts and Science, Xiangyang, China; 3 Hubei Key Laboratory of Vehicle-infrastructure Cooperation and Traffic Control, Hubei University of Arts and Science, Xiangyang, China; 4 School of Education, HuBei University of Arts and Science, Xiangyang, China; Jazan University College of Engineering, SAUDI ARABIA

## Abstract

In order to compare the effects of different anti stripping agents on the rheological properties of asphalt at high and low temperatures, four typical anti stripping agents, amine PA-1, non amine XT-2, hydrated lime, and cement, were selected. The complex modulus (G*), phase angle (δ), rutting factor (G*/sinδ), creep stiffness (S), and creep rate (m) were measured using the dynamic shear rheometer (DSR) and the bending beam rheometer (BBR). The microscopic mechanism was analyzed using fourier transform infrared spectroscopy (FTIR) and fluorescence microscopy (FM). The results showed that lime and cement significantly improved the high temperature performance of asphalt, with an average increase of 1.4 and 0.8 times in G */sinδ. However, PA and XT reduced the high temperature performance, with an average decrease of 19% and 11% in G */sinδ. PA and XT have little effect on the low temperature performance of asphalt, but lime and cement will reduce the low temperature performance, with an average increase of 64% and 49% in S. The results of FTIR and FM indicate that lime and cement undergo chemical reactions with asphalt, while PA and XT do not, but PA and XT can promote the swelling of modifiers in asphalt.

## 1 Introduction

With the development of highway construction, high-quality road building materials such as basalt that can be mined are gradually becoming scarce. Some acidic stones with high hardness and good wear resistance, such as granite and quartzite, are gradually being used in asphalt pavement. Due to the strong hydrophilicity of acidic stone surfaces, asphalt cannot adhere firmly to them. Therefore, under the combined action of moisture and vehicle load, aggregates and asphalt are easily peeled off, leading to water damage to asphalt pavement [1]. In order to improve the water damage of asphalt pavement, the addition of anti stripping agents to asphalt has gradually become one of the commonly used methods. Al-Kheetan [2] studied the use of anti stripping agents to enhance the adhesion between ceramic particles and asphalt, providing a solution for the recycling of waste ceramics. Shu [3] and Dan [4] have shown that anti stripping agents can improve the adhesion between granite and asphalt, as well as the water stability of asphalt mixtures. Zhang [5] studied the use of anti stripping agents to improve the water stability of asphalt mixtures in saline and humid environments. Liu [6] and Zhu [7] pointed out that the addition of anti stripping agent can improve the aging resistance of asphalt.

The initial anti stripping agents used were mainly inorganic, such as lime, cement, etc. Zaidi [8] evaluated the improvement effect of lime on the water damage resistance of asphalt mixtures based on surface free energy theory and adhesion tests. Kim [9] developed a granular anti stripping agent using lime as raw material, and the loss rate of Cantabro scattering test was less than 20% after adding it to asphalt mixture. Zhang [10] found that a 2% cement content can effectively improve the water damage resistance of tuff mixtures. Although inorganic anti stripping agents can improve the water stability of asphalt mixtures, they often have problems such as poor dispersibility and easy agglomeration. These inherent limitations will seriously affect their practical application in engineering and may further impair the crack resistance and fatigue performance of the mixture. In recent years, polymer based anti stripping agents have been widely used, mainly including amine and non amine types. They have the advantages of low dosage, significant improvement in adhesion, easy addition, and good compatibility with asphalt. Lucas [11] found that amine anti stripping agents not only improve the adhesion between asphalt and aggregates, but also contribute to the improvement of fatigue resistance of asphalt mixtures. Due to the easy decomposition of amine anti stripping agents at high temperatures, many non amine anti stripping agents have been developed. Al-Safar [12] used non amine anti stripping agents to improve the water stability of asphalt mixtures, and found that the freeze-thaw splitting tensile strength ratio (TSR) significantly increased from 80.6% to 94.9%. Muhmood [13] added an environmentally friendly non amine anti stripping agent to asphalt mixtures, achieving a TSR of 95%. In addition, scholars also compared the improvement effects of different anti stripping agents. Nazirizad [14] compared lime with liquid anti stripping agents and found that asphalt mixtures with added liquid anti stripping agents have better resistance to water damage. Zheng [15] compared the effects of amine and non amine anti stripping agents on the interfacial strength between asphalt and aggregate, and found that the addition of amine anti stripping agents reduced the adhesion strength, while the addition of non amine anti stripping agents enhanced the adhesion strength. Shu [16] proposed a composite anti stripping agent and found that it is more effective in improving the adhesion of asphalt compared to a single anti stripping agent. Current research mainly focuses on the influence of anti stripping agents on the adhesion between asphalt and aggregate, as well as the inhibitory effect of water damage. However, there is still insufficient comparative research on the rheological properties of asphalt at high and low temperatures using different anti stripping agents, especially amines, non amines, hydrated lime, and cement. This has an important guiding role for the application of different types of anti stripping agents in different climatic environments.

In order to systematically study the effects of different types of anti stripping agents on the rheological properties of asphalt. The DSR and BBR were adopted to systematically study the influence laws of four typical anti stripping agents (amine, non-amine, hydrated lime, and cement) on the rheological parameters of asphalt at high and low temperatures, such as G*/sinδ, S, and m. The evolution characteristics of functional groups were analyzed by combining FTIR, and the phase distribution was observed by FM to reveal the influence mechanism of different anti stripping agents on the performance of asphalt. The research results will provide theoretical support for the scientific selection of anti stripping agents in different environmental regions.

## 2 Raw materials

### 2.1 Asphalt

The asphalt used in this study is high viscosity modified asphalt, and its basic technical indicators are shown in Table 1.

**Table 1:** Technical indicators of asphalt.

Item		Unit	Requirement	Result	Test method
Penetration (25°C, 100g, 5s)		0.1mm	≥40	44.80	T0604
Softening point		°C	≥80	91.00	T0606
Flash point		°C	≥260	275	T0611
Solubility		%	≥99	99.6	T0607
Brinell viscosity (135°C)		Pa•s	–	4.308	T0625
Dynamic viscosity (60°C)		Pa•s	≥50000	103018	T0620
Elastic recovery (25°C)		%	≥95	99.24	T0662
After RTFOT	Mass change rate	%	±0.2	0.06	T0609
	Penetration residual rate	%	≥65	86.45	T0604

### 2.2 Anti stripping agent

**Fig 1:**
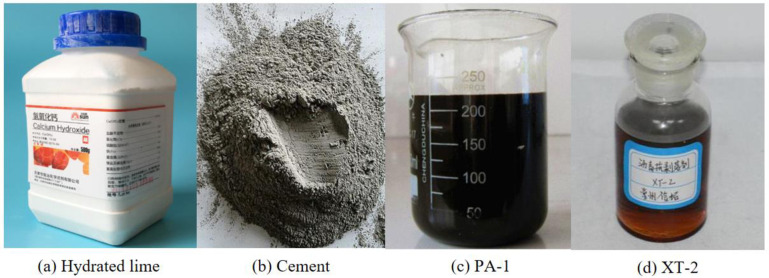
Four different anti stripping agents.

The main principle of anti stripping agent is that its alkaline components can react with the hydroxy acids in asphalt, thereby generating substances with strong adsorption capacity for aggregates, achieving the goal of improving the water stability of asphalt mixtures. Four different types of anti stripping agents were selected in this study, namely PA-1 amine anti stripping agent, XT-2 non amine anti stripping agent, hydrated lime, and cement, as shown in Fig 1.

PA-1 is an amine anti stripping agent developed by Xi’an Highway Research Institute. XT-2 is a non amine anti stripping agent developed by Changzhou Xintuo Road Surface Modified Materials Co., Ltd.‌ The parameters of PA-1 and XT-2 anti stripping agents are shown in Table 2.

**Table 2:** Parameters of PA-1 and XT-2.

Type	Appearance	Density (g/cm3)	Degradation temperature(°C)	Solubility	Recommended dosage	product type
PA-1	Brown viscous liquid	0.98	＞300	Dissolve well in hot asphalt	0.3% ~ 0.5%	Amine
XT-2		1.02			0.2% ~ 0.5%	Non amine

Grade I hydrated lime is selected, with active ingredients of calcium oxide and magnesium oxide, accounting for 68.3%, and a density of 2.69/cm^3^.

42.5 ordinary Portland cement is selected, with a 28 day compressive strength of 52.3MPa and a density of 3.128g/cm^3^.

The comparison of four anti stripping agents in terms of economy, availability, and environmental impact is shown in Table 3.

**Table 3:** Comparison of the use of four anti stripping agents.

Item	Hydrated lime	Cement	PA-1	XT-2
Cost	Low	Low	Moderate	High
Availability	Strong adaptability and can significantly improve the adhesion of acidic aggregates, such as granite.	The effect is inferior to that of hydrated lime.	Short term water stability is good, but it may be affected by thermal aging.	Good heat aging resistance, high long-term effectiveness, and wide applicability.
Environmental impact	Alkaline dust may affect the breathing of construction workers.	Production energy consumption and carbon emissions are relatively high.	Liquid dosage form, easy to use, and low odor	An environmentally friendly formula.

## 3 Experimental

Four anti stripping agents were added according to the percentage of asphalt mass in Table 4, and then the rheological and micro performance tests were conducted.

**Table 4:** Proportions of anti stripping agents.

Type	Mixing ratio/%		
Lime	20	30	40
Cement	20	30	40
PA-1	0.2	0.4	0.6
XT-2	0.2	0.4	0.6

### 3.1 High temperature rheological performance test

**Fig 2:**
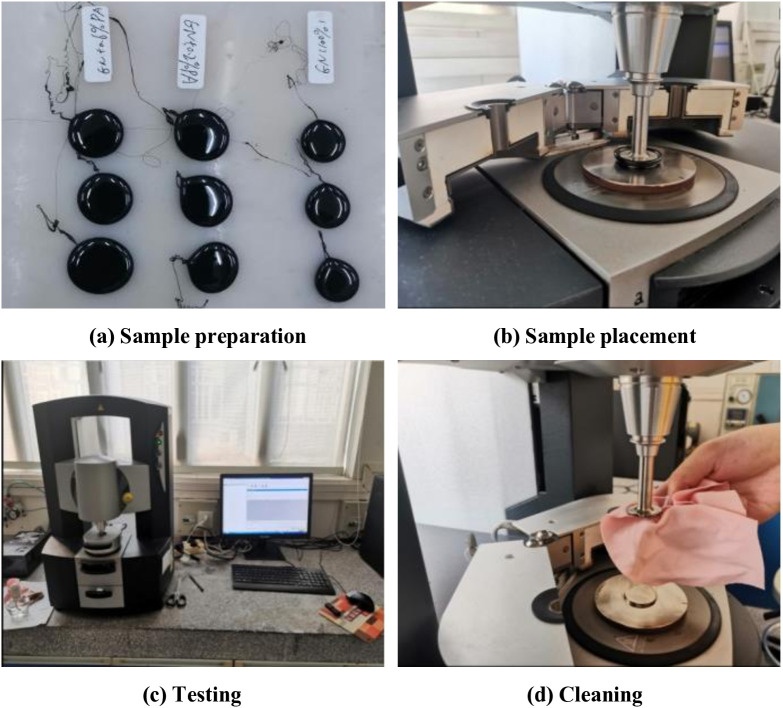
Process of DSR test.

The DSR produced by TA Company in the United States was used to conduct high-temperature dynamic shear rheological tests on asphalt samples. The experiment involved several steps, including sample preparation, sample placement, testing, and cleaning, as shown in Fig 2.

During the experiment, the asphalt sample was sandwiched between two parallel plates with a diameter of 25 mm, with a spacing of 1 mm between the plates. One plate was fixed while the other rotated back and forth around the central axis. The oscillation plate rotated at a frequency of 10 rad/s, and the test temperatures were set to 58, 64, 70, 76, 82, and 88°C in sequence. Through experiments, indicators such as phase angle δ, complex shear modulus G*, and rutting factor G */sinδ can be obtained.

### 3.2 Low temperature rheological performance test

**Fig 3:**
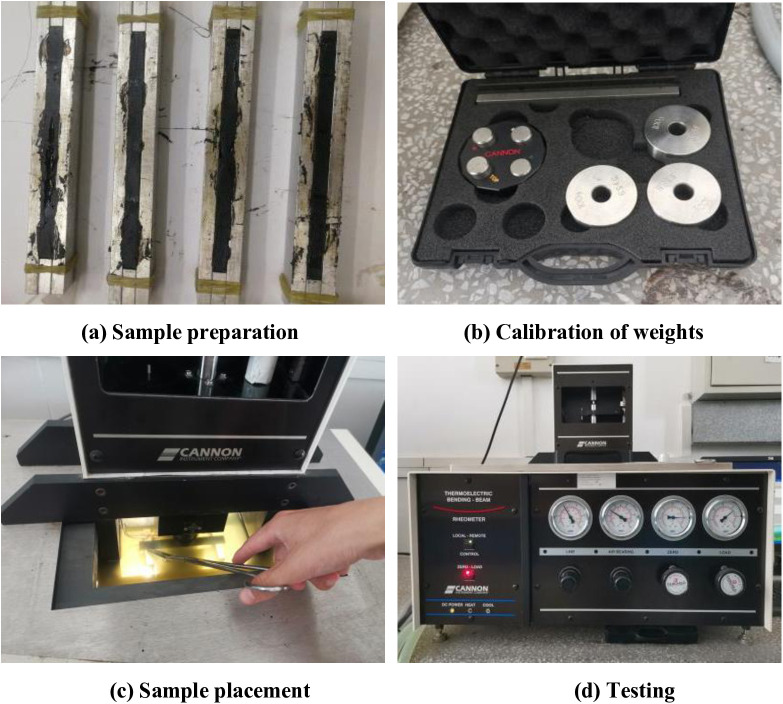
Process of BBR test.

The low-temperature rheological properties of asphalt samples were tested through the BBR test. The test involved several steps, including sample preparation, weight calibration, sample placement, and testing, as shown in Fig 3.

Asphalt beam specimens with dimensions of 127 mm in length, 12.7 mm in width, and 6.4 mm in thickness were prepared before the experiment. Then, the ethanol liquid was poured into the instrument and the temperature was adjusted to the set temperature. The temperatures for this experiment were set to −6°C, −12°C, −18°C, and −24°C, respectively. A stainless steel beam with a thickness of 6.4 mm and four weights with a mass of 100g were used to calibrate linear variable displacement transducer (LVDT) and load sensors. Finally, the asphalt beam was placed for testing, and the test data will be automatically collected by the system. During the test, a vertical load of 0.98N ± 0.05N was applied to the middle point of the beam for 240s, and the load and deformation were recorded for each 0.5s. The creep stiffness S and creep rate m at the 60th second were used to evaluate the low-temperature rheological properties of asphalt. Formula (1) and (2) were used to calculate creep stiffness S and creep rate m.


$$S(t)=\frac{Pl^{3}}{4bh^{3}\delta (t)}$$ (1)

$$m(t)=\left|\frac{d\mathrm{lg}S(t)}{d\mathrm{lg}t}\right|$$ (2)

Where: S(t) is the creep stiffness at time t, MPa; P is the fixed load, N; l is trabecular support distance, 101.6 mm; b is the beam width, 12.7 mm; h is the beam thickness, 6.4 mm; δ(t) is the beam deformation at time t, mm; m(t) is the creep rate at time t.

### 3.3 FTIR test

The AIPHA II Fourier transform infrared spectrometer produced by Bruker in Germany was used to analyze the sample. The detection wavenumber range is 600 ~ 4000 cm^-1^, the wavenumber accuracy is 2 cm^-1^, and the spectral resolution is 4 cm^-1^.

### 3.4 FM test

The distribution of anti stripping agents in asphalt was evaluated using FM test. Firstly, the asphalt sample was heated to a flowing state; Afterwards, the hot asphalt was dropped onto a glass slide and pressed into a thin layer by a cover slide; Then, it was placed in an oven and heated for about 5 minutes, followed by natural cooling; Finally, the sample can be observed using a fluorescence microscope.

## 4 Results and discussion

### 4.1 High temperature rheological properties

#### 4.1.1 Phase angle.

**Fig 4:**
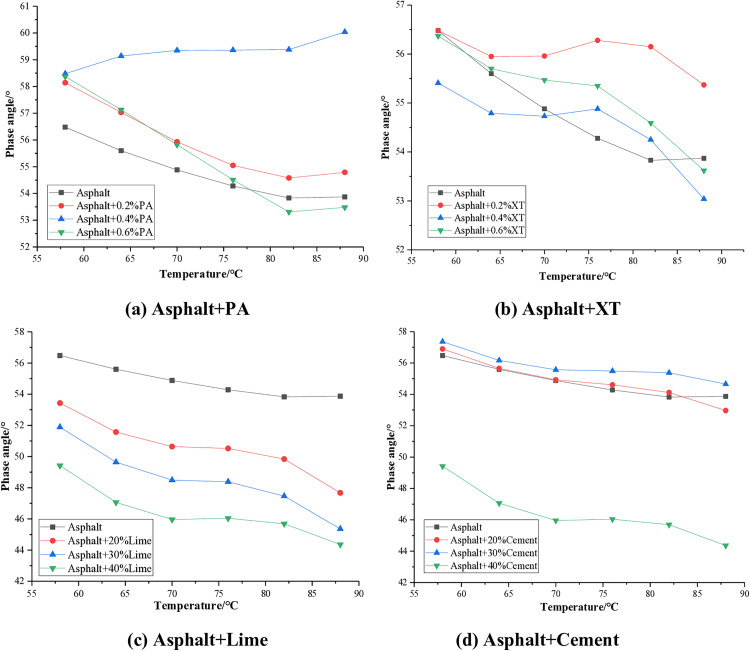
Phase angle of asphalt.

The phase angle reflects the ratio of viscosity and elastic components of asphalt binder. The larger the phase angle, the greater the viscosity component of asphalt, otherwise the more elastic component. The phase angle test results of asphalt mixed with different anti stripping agents are shown in Fig 4.

As can be seen from Fig 4, with the increase of temperature, the phase angle of asphalt basically shows a continuous decreasing trend, indicating that the proportion of the elastic part increases with the rise of temperature. The degree of phase angle reduction varies depending on the addition of different anti stripping agents. When the temperature rises from 58°C to 88°C, the average phase angle decreases by 3.8% with the addition of PA, 3.7% with XT, 11.2% with lime, and 7.3% with cement. The reduction degree of lime and cement is the greatest, indicating that they can significantly improve the elastic ratio in asphalt.

When PA is added to the asphalt, the phase angle increases and reaches its maximum at a dosage of 0.4%. When XT is added to asphalt, the phase angle also increases and reaches its maximum at a dosage of 0.2%. This indicates that the addition of PA and XT will increase the viscosity ratio of asphalt. This is mainly because both PA and XT are in liquid state and can reduce the viscosity of asphalt through the “interface lubrication effect”, resulting in an increase in phase angle. After adding lime to asphalt, the phase angle decreases, and the larger the dosage, the more it decreases. This indicates that the addition of lime can significantly increase the elastic ratio of asphalt. When a small amount of cement is added to asphalt, the phase angle does not change very much. However, when the dosage reaches 40%, the phase angle drops significantly. Just like lime, it can also enhance the elastic properties of asphalt.

#### 4.1.2 Complex shear modulus.

**Fig 5:**
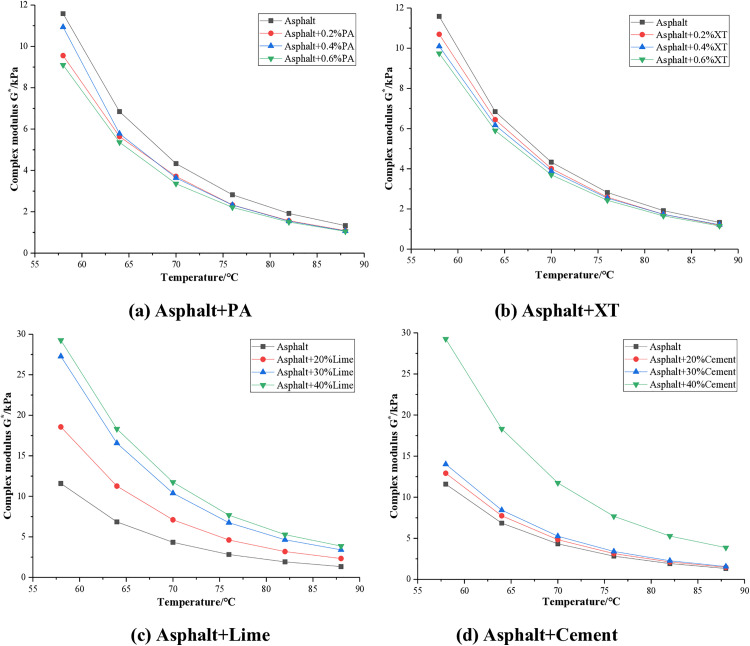
Complex modulus of asphalt.

The complex modulus G* reflects the ability of asphalt to resist deformation, and the larger its value, the stronger the ability of asphalt to resist deformation. The complex modulus test results of asphalt with different anti stripping agents are shown in Fig 5.

It can be seen from Fig 5 that the complex modulus will decrease with the increase of temperature, and the rate of decrease will be fast at first and then slow down. When PA and XT anti stripping agents are added to asphalt, the complex modulus of both decreases, and the greater the dosage, the greater the reduction. The average decrease after adding PA is about 17%, and the average decrease after adding XT is about 11%. This indicates that the addition of these two liquid anti stripping agents will reduce the deformation resistance of asphalt, which is consistent with the research conclusions of Zhu [7] on three different liquid anti stripping agents. When lime and cement are added, the composite modulus of asphalt increases. After adding lime, the complex modulus increased by an average of about 1.2 times, and after adding cement, it increased by an average of about 0.6 times. The improvement effect of lime on the deformation resistance of asphalt is more significant under the same dosage.

#### 4.1.3 Rutting factor.

**Fig 6:**
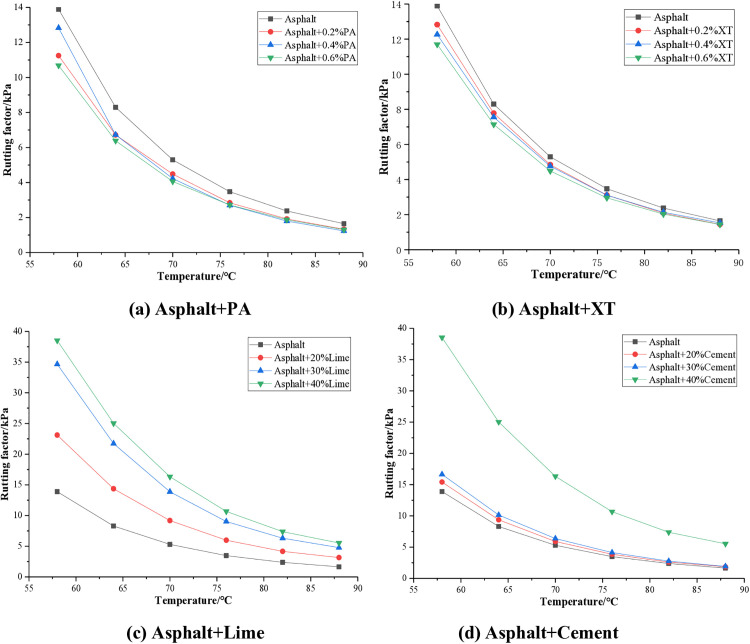
Rutting factor of asphalt.

According to G* and δ, the rutting factor G*/sinδ can be calculated, which is an indicator for evaluating the high temperature performance of asphalt. The larger the G*/sin δ, the better the high temperature performance of asphalt. The test results of asphalt rutting factor with different anti stripping agents are shown in Fig 6.

From Fig 6, it can be seen that the rutting factor of asphalt mixed with four anti stripping agents decreases with the increase of temperature. When PA and XT anti stripping agents are added to the asphalt, the rutting factor will decrease, with an average reduction of about 19% for PA and about 11% for XT. The addition of PA and XT reduces the high temperature performance of asphalt. When lime and cement are added to asphalt, the rutting factor will be improved, with an average increase of about 1.4 times for lime and about 0.8 times for cement. Lime and cement significantly enhance the high temperature performance of asphalt. From the perspective of improving the high temperature performance of asphalt, lime and cement are superior to PA and XT anti stripping agents. Lime and cement increase the high temperature performance of asphalt, while PA and XT reduce the high temperature performance of asphalt. Therefore, when constructing asphalt pavement in hot areas, it is recommended to prioritize the use of mineral anti stripping agents such as lime or cement, as they are more beneficial for the high-temperature stability of asphalt pavement.

### 4.2 Low temperature rheological properties

#### 4.2.1 Creep stiffness.

**Fig 7:**
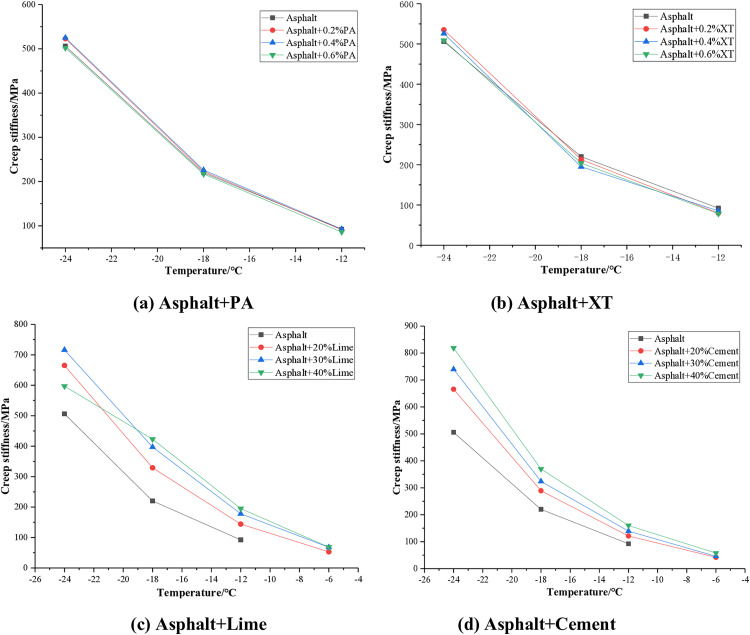
Creep stiffness of asphalt.

The creep stiffness test results of asphalt with different anti stripping agents are shown in Fig 7.

It can be seen from Fig 7 that the creep stiffness of asphalt decreases with increasing temperature. The addition of PA and XT anti stripping agents to asphalt has a relatively small effect on the creep stiffness, with a change value within 5%. The addition of lime and cement significantly increases the creep stiffness of asphalt, indicating that they weaken the low temperature performance of asphalt. After the addition of lime, the creep stiffness of asphalt increased by an average of approximately 64%, and that of cement increased by an average of about 49%. Under the same dosage, the increase of creep stiffness by lime is more significant than that by cement, which is more unfavorable for the low temperature performance of asphalt. The effects of mineral anti stripping agents and liquid anti stripping agents on the creep stiffness of asphalt show opposite characteristics, which is consistent with the results of Mario [17]. This is mainly because lime and cement can promote the formation of a more stable bonding structure in asphalt through chemical reactions and filling effects, thereby significantly increasing the creep stiffness of asphalt. However, liquid anti stripping agents such as PA and XT have surface activation effects, enhancing the fluidity of asphalt and thereby significantly reducing its creep stiffness.

#### 4.2.1 Creep rate.

**Fig 8:**
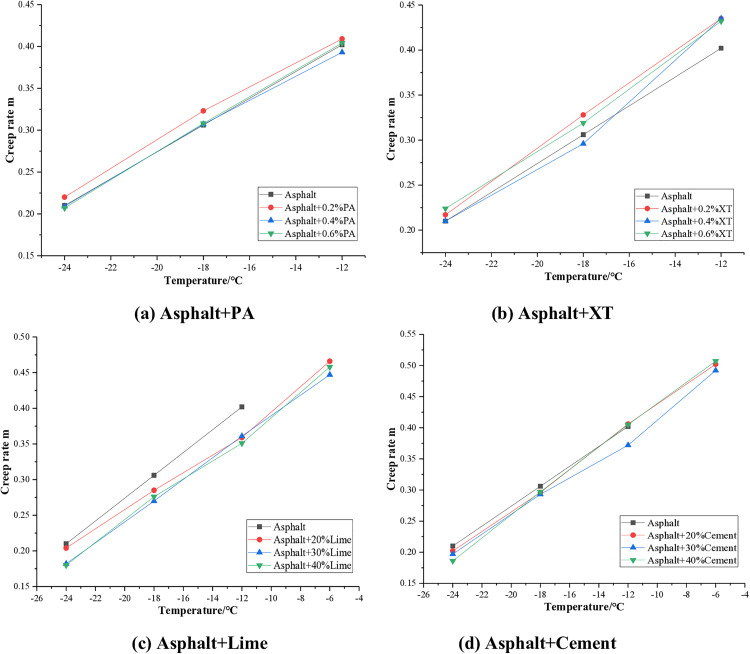
Creep rate of asphalt.

The creep rate test results of asphalt mixed with different anti stripping agents are shown in Fig 8.

It can be seen from Fig 8 that the creep rate of asphalt increases with the increase of temperature. After the addition of PA and XT anti stripping agents, the creep rate of asphalt increased to some extent, with an average increase of about 1% for PA and about 5% for XT. Indicating that PA and XT can enhance the low temperature performance of asphalt. After the addition of lime and cement, the low temperature creep rate of asphalt decreased, with an average decrease of about 10% for lime and about 4% for cement, indicating that lime is more detrimental to the low temperature performance of asphalt than cement. Therefore, when constructing asphalt pavement in relatively cold areas, it is recommended to prioritize the use of liquid anti stripping agents such as PA or XT, as they are more favorable for the low temperature performance of asphalt pavement.

### 4.3 FTIR test results

**Fig 9:**
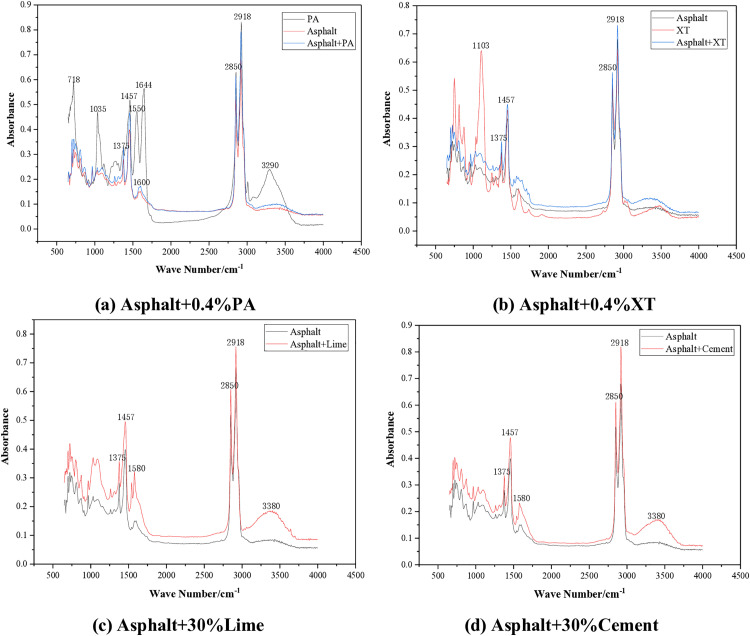
Fourier Transform infrared spectrum of asphalt.

The infrared spectra of five asphalt samples are shown in Fig 9.

It can be seen from Fig 9 that the infrared spectra of the five asphalt samples are basically similar. All samples show obvious characteristic peaks at 1375 cm-1, 1457 cm-1, 2850 cm-1 and 2918 cm-1. 1375 cm-1 belongs to the bending vibration of methyl (-CH3-). 1457 cm-1 belongs to the methylene (-CH2-) bending vibration. The peaks at 2850 cm-1 and 2918 cm-1 are respectively caused by the symmetrical stretching vibration and asymmetric stretching vibration of methylene (-CH2-).

The peaks of the amine anti stripping agent PA are 3290 cm-1, 1644 cm-1, and 1550 cm-1, which are respectively caused by the stretching vibration of -NH-, the bending vibration of -NH₂-, and the bending vibration of -NH₃ ⁺ -. After PA is added to the asphalt, the characteristic peaks of the infrared spectrum did not change significantly. The possible reason is that PA is only dispersed in the asphalt through physical adsorption and no chemical reaction occurred.

The peak of non amine anti stripping agent XT is 1103 cm-1, which is an asymmetric stretching vibration of Si-O-C or Si-O-Si, and is a characteristic peak of silane modification. After XT is added to the asphalt, there is no significant change in the characteristic peaks, indicating that no chemical reaction occurred between XT and the asphalt. PA and XT liquid anti stripping agents mainly improve the adhesion between asphalt and aggregate through physical action. The hydrophobic groups in the anti stripping agent will form a barrier to prevent water from replacing the asphalt film. Secondly, the surface active substances in the anti stripping agent can fill the tiny pores between asphalt and aggregate, forming a mechanical interlocking effect. Therefore, the high temperature of asphalt cannot be improved, but its low temperature performance can be effectively enhanced by liquid anti stripping agents.

After lime and cement are added to the asphalt, new peaks appeared at 1580 cm-1 and 3380 cm-1. The appearance of these two new characteristic peaks may indicate the occurrence of a chemical reaction. Carboxylic acids (-COOH) in asphalt undergo saponification reaction with Ca(OH)₂ to form carboxylate salts (COO^-^). The antisymmetric stretching vibration of carboxylate (COO^-^) results in a peak at 1580 cm-1. And 3380 cm-1 is caused by free hydroxyl (-OH) stretching vibration. Lime and cement mainly enhance the adhesion between asphalt and aggregate through chemical reactions. The carboxylic acid salts generated by the reaction have strong adsorption properties and can firmly adhere to the surface of the aggregate, preventing the detachment of asphalt due to water erosion. However, after the light components in asphalt are adsorbed and reacted, the asphalt becomes harder and more brittle, significantly improving its high-temperature rutting resistance and reducing its low-temperature performance.

### 4.4 FM test results

**Fig 10:**
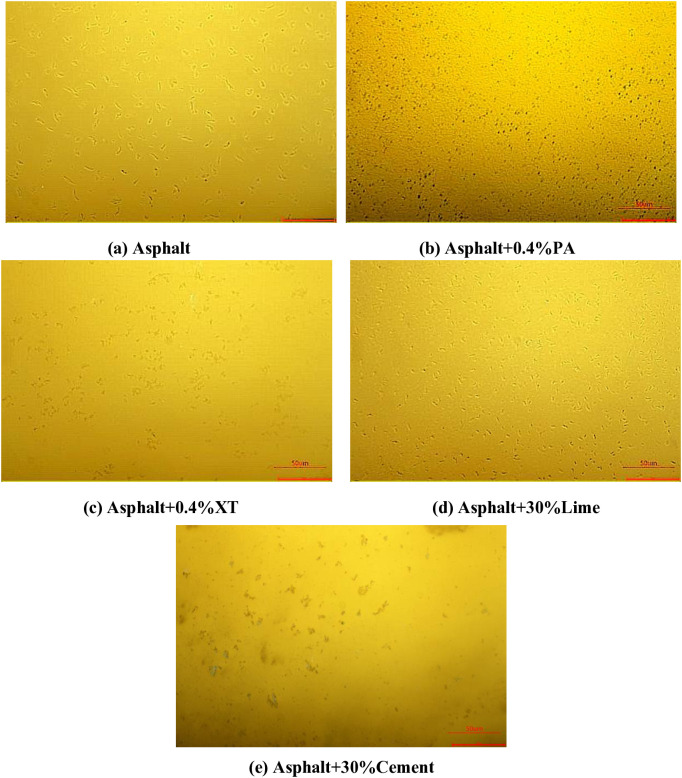
Fluorescence micrograph of asphalt.

The fluorescent microscope image of asphalt mixed with different anti stripping agents are shown in Fig 10.

It can be seen from Fig 10 that the high viscosity modifier in asphalt is evenly distributed under the fluorescence microscope. After PA and XT anti stripping agents are added to the asphalt, it can be clearly seen that the modifiers change from granular to fine flocculent. This is mainly because PA and XT anti stripping agents are liquid and can promote the swelling of the modifier. After lime and cement are added to asphalt, the dispersion state of the modifier changed little, indicating that they can not promote the swelling of the modifier.

## 5 Conclusion

In this study, the effects of four typical anti stripping agents (PA, XT, lime, and cement) on the rheological properties of asphalt at high and low temperatures and microscopic mechanisms were analyzed by DSR, BBR, FTIR, and FM. The main conclusions are as follows:

(1) Lime and cement significantly improved the rutting resistance of asphalt at high temperatures, with an average increase of 1.4 and 0.8 times in rutting factor, respectively. Conversely, PA and XT impaired this performance, reducing the rutting factor by 19% and 11%.
(2) The low temperature performance was degraded by lime and cement, as evidenced by an average increase in creep stiffness of 64% and 49%, alongside a decrease in creep rate of 10% and 4%, respectively. PA and XT exhibited negligible effects, indicating their greater applicability in cold regions.
(3) FM observation showed that PA and XT promoted the swelling of modifiers in asphalt, but no chemical reaction was detected by FTIR. And lime and cement reacted chemically with asphalt to form carboxylate salts, and FTIR detection showed new peaks at 1580 cm-1 and 3380 cm-1.

In summary, the selection of anti stripping agents requires a balance between high and low temperature performance requirements. Prioritize lime or cement in high temperature and heavy-duty areas; In cold regions, liquid anti stripping agents PA or XT are better choices. This study provides a theoretical basis for the scientific selection of anti stripping agents in different climate zones.
